# Molecular Characterization of the Middle Ear Microbiome in Pediatric Otitis Media with Effusion: Diagnostic and Clinical Implications

**DOI:** 10.3390/jcm15114200

**Published:** 2026-05-29

**Authors:** Maciej Szwajkowski, Jagoda Szwach, Sara Shefa, Anna Karwowska, Aleksandra Głębocka, Katarzyna Pazdro-Zastawny, Karolina Dorobisz

**Affiliations:** 1 Student Scientific Association of Otolaryngology, Head and Neck Surgery, Wroclaw Medical University, 50556 Wrocław, Poland; jagoda.szwach@student.umw.edu.pl (J.S.); sara.shefa@student.umw.edu.pl (S.S.); anna.karwowska@student.umw.edu.pl (A.K.); aleksandra.glebocka@student.umw.edu.pl (A.G.); 2 Department of Otolaryngology, Head and Neck Surgery, Wroclaw Medical University, 50556 Wrocław, Poland; katarzyna.pazdro-zastawny@umw.edu.pl (K.P.-Z.); karolina.dorobisz@umw.edu.pl (K.D.)

**Keywords:** otitis media with effusion, pediatric otolaryngology, middle ear microbiome, 16S rRNA gene sequencing, next-generation sequencing, microbiome dysbiosis, biofilm, adenoid hypertrophy, probiotics

## Abstract

**Background:** Otitis media with effusion (OME) is a highly prevalent pediatric condition and a leading cause of conductive hearing loss in children. Its pathogenesis remains uncertain, and diagnostic and therapeutic challenges make management difficult. **Objectives:** This review evaluates current evidence on the middle ear microbiome in pediatric OME, focusing on the diagnostic value of 16S ribosomal ribonucleic acid (16S rRNA) gene sequencing and its potential clinical implications. **Methods:** A literature review was conducted using the PubMed database, including studies published between 2006 and 2026. Eligible studies involved pediatric patients with OME and examined the sources and characteristics of microbiota potentially involved in disease pathogenesis. Microbiome analysis was performed using next-generation sequencing (NGS) techniques. **Results:** Growing evidence indicates that OME is associated with microbial dysbiosis and biofilm formation rather than a sterile inflammatory process. The most frequently detected genera include *Haemophilus*, *Moraxella*, *Streptococcus*, and *Alloiococcus*, although substantial variability exists between studies. Pathogens are believed to reach the middle ear through the Eustachian tube from two main reservoirs: the nasopharynx and the adenoids. The potential role of *Helicobacter pylori* infection and gastroesophageal reflux disease (GERD) in OME pathogenesis remains uncertain and requires further investigation. NGS methods, including 16S rRNA sequencing, demonstrate higher sensitivity than conventional culture techniques, enabling the detection of fastidious and previously unrecognized microorganisms. Evidence also highlights the limited effectiveness of antibiotic therapy in OME, the persistent issue of antibiotic overuse, and the relative advantages of conservative management and microbiome-modulating approaches compared with antibiotics and surgical interventions. **Conclusions:** Current evidence suggests that OME is closely associated with microbiota dysbiosis and bacterial biofilm formation. Given the limited efficacy of antibiotics, microbiome-focused strategies—particularly probiotics—should be further explored. Molecular diagnostic methods, especially NGS, show clear advantages over traditional culture-based techniques. Future research should further evaluate microbiome modulation as a potential adjunctive or preventive strategy.

## 1. Introduction

Otitis media with effusion (OME) is a middle-ear effusion without the perforation of the tympanic membrane and leads to hearing loss without manifestations of an acute ear inflammation such as fever or otalgia [1]. In children manifestations of OME include hearing loss and aural fullness [2]. While the prevalence of otitis media (OM) in children in the world has increased, the disease severity of OM has decreased [3]. Nowadays infection is considered the main cause of OME. Immune complexes, endotoxins, viruses, and bacteria (*Streptococcus pneumoniae*, *Haemophilus influenzae*, and *Moraxella catarrhalis*) have been identified in the middle ear effusion (MEE) of patients with OME [1]. Dysfunction of the eustachian tube is a risk factor for the development of OME as it causes impaired ear fluid drainage and inflammation caused by a bacterial infection [4]. Other OME risk factors include respiratory tract infection, craniofacial malformation, laryngopharyngeal reflux, and an immune response [5]. Adenoid hypertrophy is a frequent risk factor in children with an incidence rate of OME reaching 34.98% [6]. Biofilm formation in adenoids plays a greater role in OME than mechanical obstruction [1]. For children with OME who are not at risk, such as pathological changes to the eardrum, watchful waiting and conservative treatment are advised. Nonetheless in severe cases tympanostomy tube insertion is recommended [2]. Although a healthy middle ear was presumed to be sterile, NGS allows detecting bacteria in low bacterial load body sites, including the middle ear. Novel otopathogens may be detected by 16S rRNA gene sequencing [7].

The aim of this review is to present current and emerging diagnostic methods of analyzing the middle ear microbiome such as 16S rRNA and their application in managing OME in children.

## 2. Materials and Methods

This narrative review was based on a targeted search of the literature. PubMed/MEDLINE was searched for peer-reviewed publications from 2006 to 2026, with emphasis on recent studies from 2023 to 2026. The search focused on pediatric otitis media with effusion (OME), middle ear and adenoid microbiome, 16S rRNA gene sequencing, next-generation sequencing, biofilm formation, adenoid hypertrophy, Eustachian tube dysfunction, and microbiome-modulating approaches.

The main Boolean search string used was: (“otitis media with effusion” OR OME OR “secretory otitis media”) AND (child OR pediatric OR paediatric* OR preschool OR adolescent*) AND (microbiome OR microbiota OR dysbiosis OR biofilm OR “16S rRNA” OR “16S rRNA gene sequencing” OR “next-generation sequencing” OR NGS OR adenoid* OR “adenoid hypertrophy” OR “Eustachian tube dysfunction” OR probiotic*). Additional relevant publications were identified from reference lists of selected articles and clinical guidelines.

Priority was given to original pediatric clinical studies, microbiome studies analyzing middle ear effusion, adenoid tissue, nasopharyngeal or related upper-airway samples, as well as systematic reviews, meta-analyses, Cochrane reviews, and clinical guidelines. Case reports, small case series, conference abstracts, letters, editorials, opinion articles, non-peer-reviewed publications, and studies focused exclusively on acute otitis media without effusion were not included in the main narrative synthesis. Therefore, due to substantial methodological heterogeneity across included microbiome studies, including differences in sample type, 16S rRNA region, sequencing platform, laboratory workflow, bioinformatic pipeline, and reporting metrics, the findings were synthesized descriptively. The final tables were intentionally simplified and organized qualitatively to highlight recurrent microbial patterns without pooling non-comparable quantitative data.

This review was not designed as a systematic or scoping review; therefore, no protocol was registered, no PRISMA or PRISMA-ScR flow diagram was prepared, no formal risk-of-bias assessment was performed, and no complete screening and exclusion log was maintained.

## 3. Current Management of Pediatric OME

### 3.1. The Natural Course of OME

OME is one of the most common pediatric conditions with approximately 80% of preschool-age children experiencing at least one episode [8]. OME is often a self-limiting condition, with a high rate of spontaneous resolution; therefore, surgical intervention is required only in selected patients, making the identification of appropriate candidates a primary clinical challenge [9]. Watchful waiting is initially preferred due to the high probability of spontaneous fluid clearance in the first 3 months, avoiding unnecessary exposure to the risks of general anesthesia and surgery [10].

### 3.2. Pharmacological Treatments

According to the 2023 guidelines of the National Institute for Health and Care Excellence (NICE), pharmacological treatments, including antibiotics, oral or nasal corticosteroids, antihistamines, leukotriene receptor antagonists, mucolytics, proton pump inhibitors and anti-reflux medications, or decongestants, should not be used in the treatment of OME in children under 12 years of age [11]. Mulvaney et al. conducted a comprehensive meta-analysis of 19 studies involving a total of 2581 participants aged 6 months to 12 years. Among children receiving antibiotics versus placebo or antibiotics versus no treatment, no statistically significant benefit of antibiotics was observed in terms of faster hearing improvement or long-term impact on the course of OME [12]. Nevertheless, in everyday clinical practice, antibiotics continue to be overused despite the absence of clinical indications. Roditi et al. conducted an analysis of 1,390,404,196 pediatric visits in which the study population consisted of children without acute or nonspecific otitis media. The results showed that oral antibiotics were prescribed in 32% of visits where OME was diagnosed [13].

### 3.3. Surgical Interventions

According to the 2023 Polish National Guidelines, tympanostomy should not be performed in children with a single OME episode lasting for less than 3 months after its onset or diagnosis as the fluid in the tympanic cavity can be temporarily caused by an infection and resolve spontaneously. However, bilateral tympanostomy should be recommended in patients with bilateral OME and recorded hearing disorders. To meet these criteria, hearing loss should be greater than or equal to 30dB and present for over 3 months. Furthermore, surgical intervention is strongly indicated in cases of chronic tympanic membrane retraction.

Additionally, tympanostomy tube insertion can be performed in children with unilateral or bilateral OME persisting for over 3 months with unilateral deafness, hearing loss and absence of directional hearing, as these symptoms can negatively impact child’s development [10]. The 2023 NICE guidelines recommend considering the use of grommets (tympanostomy tubes) for the management of OME-related hearing loss in children, with possible adenoidectomy afterwards [11].

Weng et al. conducted a retrospective analysis of data from 145 children with OME who underwent surgical treatment. The results demonstrated that children treated with combined tympanostomy tube insertion and adenoidectomy showed significantly better clinical outcomes at three-month follow-up compared with those treated with adenoidectomy alone. Surgical interventions have been shown to reduce local inflammatory responses and the risk of OME recurrence [14]. The 12-month follow-up study by Skarzyńska et al. additionally demonstrated the superior outcomes of surgical treatment (Insertion Ventilation Tubes and Adenoidectomy) compared with non-surgical management (Watchful Waiting Approach), as reflected by a statistically significant (*p* < 0.001) increase in the number of healthy days—with mean values of 328.0 days and 169.2 days, respectively [15]. Findings from this and other independent studies [16,17,18] indicate ongoing efforts of clinical application and the marked superiority of surgical approaches over pharmacological methods in the treatment of OME.

## 4. The Middle Ear Microbiome and 16S rRNA as a Novel Diagnostic Approach in Otitis Media with Effusion (OME)

### 4.1. The Role of NGS and 16S rRNA in Analyzing the Middle Ear Microbiome

The NGS method is a modern DNA sequencing technique that enables comprehensive analysis of genetic material. In the context of microbiome identification and characterization, sequencing of the gene encoding the 16S ribosomal subunit is particularly important. This fragment is present in all bacteria and contains both conserved regions (shared across bacterial species) and variable regions (allowing for species-level differentiation) [19,20]. Study [7] demonstrated that standard culture fails to detect fastidious (difficult-to-culture) bacteria. For instance, in patient P2, culture detected Micrococcus luteus, which constituted less than 1% of the bacterial relative abundance (i.e., the proportion of a given bacterium within the total microbial community) shown by sequencing, whereas 16S rRNA analysis revealed that the dominant bacterial genus was *Alloiococcus* (92%). Across the entire study, the prevalence of *Alloiococcus* was 97% of samples, with a relative abundance of 39%, while this strain was not detected at all in traditional culture. Standard culture methods are time-consuming and allow for the cultivation of individual bacterial strains, whereas 16S rRNA sequencing facilitates analysis of the entire microbiome in a short time, enabling a more precise assessment of the relationships between specific bacterial species [7,21]. Nonetheless, 16S rRNA next-generation sequencing (16SNGS) solely identifies the infecting pathogen; therefore, antibiotic sensibility testing must be performed to prescribe an appropriate antibiotic. Furthermore, 16SNGS is based on short reads, as it is performed on one of the variable regions of the gene. While short reads from NGS platforms exhibit higher accuracy, long-read sequencing technologies are associated with a better taxonomic classification at the genus and species level. Some bacteria might exhibit high similarity to other members of the same family, even within the variable regions of 16S rRNA sequences. Here, further sequencing of other genes will enable accurate identification. Patient medical history and clinical judgment is necessary in the proper diagnosis as the detected organism might not be the cause of the illness [22]. The detection of bacteria does not show its viability or activity [23,24]. DNA extraction kits and other laboratory reagents may be a source of contamination. During each lot and each sample processing, laboratories should determine contaminants. Instead of unprocessed water, extraction blanks should be used as a no template and other controls [25,26].

**Figure 1:**
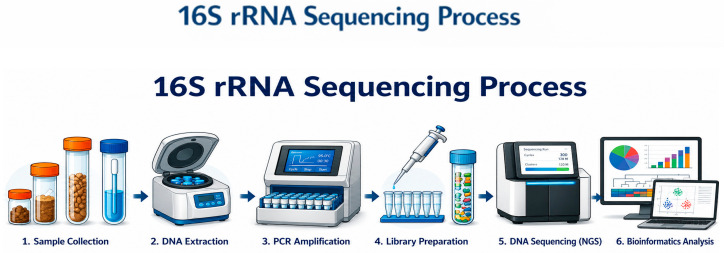
Overview of the 16S rRNA sequencing workflow for microbiome analysis.

The above infographic (Figure 1) presents a simplified schematic of microbiome sequencing using the 16S rRNA technique, which comprises the following steps:

1. Sample Collection—Collect samples such as stool, oral swabs, nasal swabs, or other biological materials containing microbial communities.
2. DNA Extraction—Isolate total DNA from microbial samples through lysis, purification, and removal of inhibitors.
3. PCR Amplification—Amplify the 16S rRNA gene using PCR primers targeting conserved regions flanking variable regions of the gene.
4. Library Preparation—Add sequencing adapters and unique barcodes to PCR products, then pool, clean, and quantify the DNA library.
5. DNA Sequencing (NGS)—Sequence the amplicons on a NGS platform such as Illumina MiSeq, generating millions of reads.
6. Bioinformatics Analysis—processing sequencing data to identify and compare bacterial taxa within the sample.

### 4.2. Does the Healthy Middle Ear Possess a Microbiome?

In the context of microbiome research, it is important to distinguish between a resident microbiome, defined as a stable and consistently detectable microbial community, and transient microbiota, which are intermittently present and often reflect migration or contamination from adjacent anatomical sites.

Based on current evidence, the microbial signals detected in the healthy middle ear are more consistent with transient microbiota rather than a true resident core microbiome. New studies employing quantitative polymerase chain reaction (qPCR) and 16S rRNA gene sequencing demonstrated that the middle ear contains an extremely low bacterial load. The level of bacteria did not significantly differ from negative control samples (*p* > 0.05), suggesting the absence of stable microbial colonization. The detection of bacteria such as *Pseudomonas*, *Methylobacterium* and *Ralstonia* most likely resulted from contamination of the sequencing reagents [27]. A recent study found a higher abundance of Ralstonia in female patients, suggesting that hormonal differences may influence microbial composition, although no consistent association with age or microbial diversity has been confirmed. Current evidence therefore suggests that microorganisms detected in the middle ear most likely reflect transient contamination or migration from adjacent anatomical sites rather than the presence of a true resident microbiome [27].

### 4.3. Sources of Bacteria in OME: A Pathogenesis Model

Multiple sequencing studies strongly support that the bacteria detected during otitis media with effusion must originate from an external source, primarily the nasopharynx, via the Eustachian tube [28]. Reservoir-focused analyses also suggest a dual-input model: classic otopathogens track with adenoid/nasopharynx, while external auditory canal-associated genera (e.g., *Alloiococcus*/*Staphylococcus*) may contribute to middle ear fluid (MEF), particularly when tympanic membrane integrity has been disrupted (e.g., prior tubes/perforation) [29].

Furthermore, Eustachian tube dysfunction occurs more frequently in children and is related to the fact that the tube is shorter and more horizontal, which contributes to an increased risk of developing otitis media with effusion and other middle ear diseases [5].

Respiratory viruses are linked to pediatric chronic OME. Runge et al. have reported the presence of respiratory viruses—most commonly rhinovirus, parainfluenzavirus and bocavirus—in the middle ear effusions of 44 out of 69 children with chronic OME [30]. Similarly, in a study by Rezes et al. Viral nucleid acid was found in 26 out of 75 middle ear effusion samples with human enteroviruses being present in 22 samples, human rhinoviruses in 10 and human bocavirus in 2 [31]. Mycobiome may participate in the pathogenesis of OME as *Candida glaebosa*, *Candida cretensis*, *Aspergillus ruber*, *Penicillium desertorum*, and *Rhizopus arrhizus* were more abundant in patients with OME [32].

The nasopharynx is considered the principal source of otopathogens. Walker et al. demonstrated that children with OME exhibit significantly greater nasopharyngeal colonization by otopathogens, with microbiome profiles dominated by *Corynebacterium*, *Streptococcus*, or *Moraxella*, whereas healthy children exhibited a more mixed microbial profile with greater abundance of commensals, including alpha-haemolytic *Streptococci* and *Lactococcus* [24]. These findings support the concept that nasopharyngeal dysbiosis plays a central role in OME development.

Kielbik et al. have reported that the introduction of the pneumococcal conjugate vaccine (PCV) in the national immunization schedule in Poland led to a significant decrease in nasopharyngeal carriage of PCV serotypes and resistant strains among vaccine serotypes and to a modest increase in non-vaccine pneumococcal serotypes carriage [33]. Salgado et al. have reported that the 10-valent PCV (PCV10) vaccination stimulates target-specific response against pathogens and leads to commensal bacteria colonization without significantly altering the nasopharyngeal microbiome. Yet it was reported that the commensal bacteria are associated with disease protection [34]. Futhermore Toizumi et al. observed a correlation between PCV10 introduction and reduced OME prevalence in infants, while not in older children [35].

Growing evidence further highlights the important correlation of chronic middle ear effusion and adenoids as a persistent microbial reservoir. Research indicates that the microbiome of chronic otitis media with effusion is significantly associated with changes in the adenoid microbiome (r^2^ = 0.097, *p* < 0.05) [4]. Adenoid microbiome studies report reduced diversity and lower operational taxonomic unit (OTU) richness in OME, where OTUs (i.e., groups of closely related bacteria used as a proxy for microbial diversity) indicate a relative enrichment of *Haemophilus* and other genera, consistent with the “bacterial interference” concept (loss of commensals leads to reduced colonization resistance) [36].

In the study by Jervis-Bardy et al., microbiome sequencing of MEF, adenoids, and the nasopharynx was performed in 11 children using 16S rRNA gene sequencing. The shared components of the microbiome across all sample types were OTUs corresponding to *Streptococcus* sp., *H. influenzae*, and *Moraxella catarrhalis*, which, consistent with previous studies, indicates the significant otopathogenic potential of these bacterial species [37].

Direct microbiological overlap between adenoidal tissue and middle ear effusion has also been demonstrated, with shared detection of species including *Alloiococcus otitidis*, *Streptococcus pneumoniae*, *Haemophilus influenzae*, and *Moraxella catarrhalis* in pediatric OME patients. These findings provide evidence supporting bacterial migration from the nasopharynx and adenoids to the middle ear via the Eustachian tube [38,39].

On the other hand, the study by Sokolovs-Karijs et al. did not demonstrate a significant difference in the bacterial composition of the adenoids between healthy patients and those with OME, suggesting a less significant contribution of the adenoid microbiome to the pathogenesis of OME [40]. However, considering numerous scientific reports, the role of the adenoids in the pathogenesis of OME cannot be overlooked.

In addition to nasopharyngeal sources, similarities between microbial communities of middle ear effusion and the external auditory canal have been reported. Dominance of genera such as *Alloiococcus* and *Staphylococcus* in both sites suggests that external auditory canal microbiota may contribute to middle ear colonization, particularly in cases of tympanic membrane disruption. Observed co-occurrence patterns among otopathogens further support a polymicrobial disease model compatible with multispecies biofilm formation [29].

**Figure 2:**
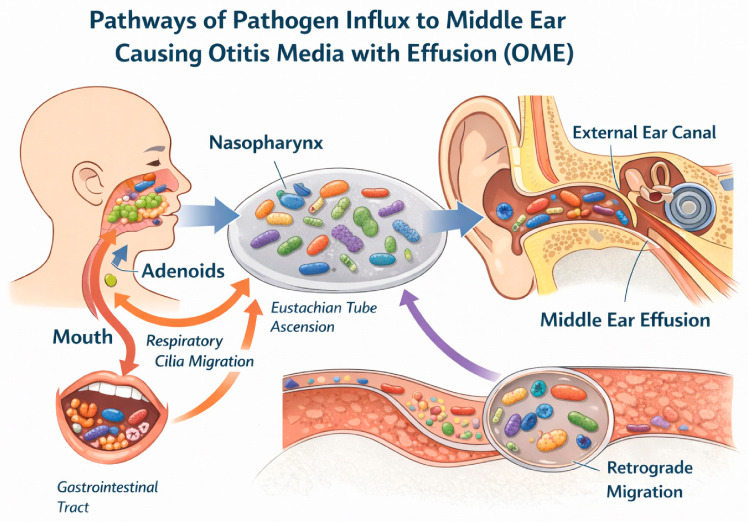
Schematic representation of pathogen influx pathways to the middle ear. Arrows indicate the proposed routes of pathogen migration, including ascent from the nasopharynx through the Eustachian tube, respiratory cilia-mediated movement, gastrointestinal/oral contribution, and retrograde migration. Different studies have examined the relationship between the presence of *Helicobacter pylori* and the development of OME. In studies conducted by B. V. Agirdir and M. A. Damghani, *H. pylori* was detected in middle ear fluid in as many as 66% to 70% of patients [44,45]. In addition, the study by D. Mel-Hennawi showed that the presence of *H. pylori* affects the results of standard OME treatment, and its eradication significantly improved treatment outcomes [46].

Emerging evidence also suggests a potential contribution of oral microbiota. Genetic similarity between *Fusobacterium* nucleatum strains identified in saliva, nasopharyngeal samples, and middle ear effusion indicates that translocation from the oral cavity may occur in selected patients, although this pathway likely represents a secondary mechanism requiring further investigation [41]. Another study demonstrated a statistically significant association between the occurrence of OME and early childhood caries in preschool children [42]. In turn, the study by Jalali MM et al. demonstrated that the risk of middle ear effusion in children with dental caries was 139% higher than in children without caries [43]. These findings further support the hypothesis that oral pathogens may contribute to the pathogenesis of OME in pediatric patients. Taken together, these findings support a multifactorial model of pathogen influx to the middle ear, involving nasopharyngeal and adenoidal reservoirs, Eustachian tube ascent, possible oral or gastrointestinal contribution, and retrograde migration, as summarized schematically in Figure 2.

However, in another study conducted by using advanced molecular sequencing techniques, the authors did not detect *H. pylori* in any samples obtained from the middle ear or adenoids, either in children with OME or in healthy controls [47]. Similarly, in a study by A. Jeyakumar et al., H. pylori was not identified in any samples using 16S rRNA sequencing [48,49,50].

On the other hand, a study involving a group of 45 patients conducted by T. Fancy et al. showed that although *H. pylori* may be present in the nasopharynx and middle ear of children, there is no significant correlation between its presence and the occurrence of OME [51]. The studies indicate discrepancies in the results and conclusions regarding the significance of *H. pylori* in the pathogenesis of OME, highlighting the need for further data in future research.

Previous studies have also indicated a significant association between the coexistence of GERD and OME in children [48,49,50]. In contrast, Boers et al. have reported that GERD has no obvious impact on nasopharyngeal and middle ear microbiota. In this study among 23 pediatric patients with chronic OME, no upper gastrointestinal tract microbiota once linked to OME was detected in the middle ear. Furthermore, patients with or without GERD did not have any difference in the microbiota of the middle ear fluid [52].

### 4.4. Characterization of the Microbiome in Pediatric OME

Clinical studies indicate that bacteria of the genera *Haemophilus*, *Moraxella*, *Streptococcus*, *Alloiococcus*, and *Turicella* play a central role in the pathogenesis of OME. While some studies report a significant dominance of a single bacterial species, others describe mixed microbial communities. However, numerous sources in the medical literature report a variety of other, less frequently detected pathogens in OME. This observation, together with current knowledge regarding the potential migration of bacteria to the middle ear, supports the hypothesis that there is no universal microbiome composition in OME [4,53,54]. Given the low biomass of middle ear samples, these findings require cautious interpretation due to potential contamination. Distribution of the main microorganisms associated with OME is presented in Table 1.

Given the substantial heterogeneity in sequencing regions, sampling strategies, and bioinformatic pipelines across included studies, bacterial taxa were summarized qualitatively rather than pooled quantitatively. Table 2 presents the genera most consistently reported across pediatric OME microbiome studies and indicates their typical anatomical source, reproducibility across studies, and putative clinical relevance.

**Figure 3:**
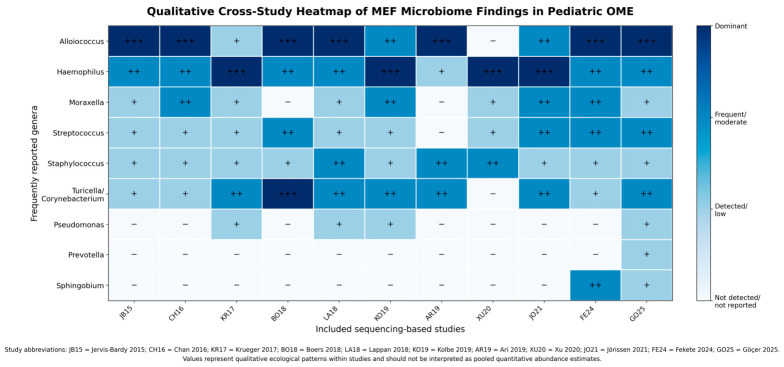
Qualitative cross-study heatmap of recurrent bacterial genera reported in pediatric otitis media with effusion (OME) microbiome studies. The heatmap summarizes middle ear effusion/middle ear cavity sequencing-based studies included in the qualitative synthesis: Jervis-Bardy et al. 2015 [37], Chan et al. 2016 [29], Krueger et al. 2017 [55], Boers et al. 2018 [52], Lappan et al. 2018 [23], Kolbe et al. 2019 [54], Ari et al. 2019 [38], Xu et al. 2020 [56], Jörissen et al. 2021 [57], Fekete et al. 2024 [7], and Göçer et al. 2025 [60]. The heatmap uses a structured qualitative scale: +++, dominant within the study; ++, frequently reported or moderately abundant; +, detected at low abundance; and −, not detected or not reported. Signal intensity reflects qualitative dominance within individual studies and does not represent pooled or averaged relative abundance. Due to substantial methodological heterogeneity between studies, the figure should be interpreted as an illustrative overview of recurrent microbial patterns rather than a quantitative meta-analysis.

To provide a transparent synthesis of the heterogeneous microbiome literature, findings were organized according to anatomical niche and study type rather than pooled into a single quantitative estimate. Table 3, Table 4, Table 5, Table 6 and Table 7 summarize middle ear, adenoid, nasal/nasopharyngeal, biofilm-related, and methodological findings separately, allowing recurrent microbial patterns to be interpreted in their appropriate biological and technical context. This structure avoids conflating relative abundance, detection frequency, and culture/PCR positivity while preserving clinically relevant information on OME-associated microbial ecology. To further visualize the recurrent qualitative patterns of bacterial genera reported across middle ear effusion and middle ear cavity microbiome studies, these findings are summarized in Figure 3.

## 5. Clinical Potential Arising from Discoveries Enabled by Molecular Diagnostic Techniques

The potential for modulating the middle ear and adenoid microbiota toward a non-pathogenic, healthy state warrants investigation as a strategy to reduce OME incidence and limit the need for invasive surgical interventions.

Jörissen et al. conducted a groundbreaking case–control study in which the 16S rRNA method was used to analyze the microbiome of 70 children with chronic OME and 53 healthy controls. The aim of the study was not to identify potentially pathogenic microorganisms, but rather those that might exert a preventive effect against the development of OME. A key component of the study was the evaluation of 79 isolates representing 13 *Streptococcus* species for their pathobiont-inhibiting activity. *Streptococcus salivarius* demonstrated the most significant effect, showing the strongest in vitro inhibition of *H. influenzae*, *Moraxella catarrhalis*, *Streptococcus pneumoniae*, *Streptococcus pyogenes*, *Staphylococcus aureus*, *A. otitis*, and *Corynebacterium otitidis* [57].

This approach is consistent with the concept of bacterial interference discussed in the otitis media literature, in which commensal organisms that naturally occupy the upper respiratory tract are used to outcompete otopathogens. In vivo studies have shown that bacterial interference occurs through the presence on specific sites on epithelial cells in order to prevent the adherence of pathogens. Other mechanisms involve modification of the microenvironment by producing antagonistic substances, lowering the pH and competition for nutrients. Reviews emphasize that targeting niche-specific strains with demonstrated inhibitory activity is likely to be more effective than administering general probiotic blends [64].

The ability of *S. salivarius* to suppress multiple otopathogens suggests that carefully selected probiotics might help restore a healthier nasopharyngeal microbiota and thereby contribute to both prevention and treatment of chronic OME. Initial clinical evidence supports this idea, particularly for topical administration. In a double-blind, randomized pilot trial in children with chronic secretory otitis media, an intranasal spray containing *Streptococcus sanguinis* or *Lactobacillus rhamnosus* was tested. After 10 days, 7 of 19 children receiving *S. sanguinis* were completely or significantly recovered, compared with 1 of 17 on placebo (*p* = 0.044), whereas the *L. rhamnosus* spray had no significant benefit over placebo [65].

More recently, a placebo-controlled trial in 3–6-year-old children with adenoid hypertrophy and OME who were receiving conservative management tested a daily lozenge containing *S. salivarius* K12 (SSK12) (≥10^9^ CFU). In vitro models suggest that SSK12 may suppress key pathogens and decrease local inflammation, mucus viscosity and enhance ciliary clearance. After 12 weeks, tympanogram normalization (Jerger type B/C to A) was seen in 50.4% of children in the SSK12 group versus 35.7% in the placebo group. Adjusted analyses yielded an odds ratio of 1.87, and the probiotic group also showed higher colonization by the probiotic strain, lower loads of *H. influenzae*, *S. pneumoniae* and *M. catarrhalis*, and improved hearing parameters, with no notable safety issues [66]. These early clinical results suggest potential benefits; however, widespread clinical recommendation requires further validation.

Nevertheless, not all probiotic interventions yield clear benefits. In another randomized double-blind trial, children awaiting tympanostomy received oral *Lactobacillus rhamnosus* GG (LGG) for three weeks. Although LGG DNA was detected in some middle-ear effusion samples, there was no reduction in the prevalence of bacterial or viral pathogens, indicating that mere detection of a probiotic in the middle ear does not guarantee clinical or microbiological efficacy [67].

Taken together, these studies indicate that further progress will depend on rigorous placebo-controlled human trials that use objective OME endpoints such as tympanometric classification, duration of effusion and hearing assessments alongside microbiological readouts of colonization and pathogen load. Such work is essential to determine which probiotic strains, dosages and delivery routes yield meaningful clinical benefits in the management of OME. From a broader perspective, the global burden of otitis media (OM) in children remains a highly significant public health issue. In 2021 alone, more than 297 million cases were reported worldwide among individuals aged 0–14 years [68]. Additionally, next-generation sequencing (NGS) methods, including 16S rRNA sequencing, remain substantially more expensive than many other molecular diagnostic tests [69,70]. Although the upfront costs of molecular diagnostics are higher, their use in selected cases to analyze and expand knowledge of the specific microbiota involved in the pathogenesis of OME may contribute to long-term cost savings by reducing ineffective treatments, minimizing complications, and decreasing the need for repeated medical interventions. Future studies should evaluate the cost-effectiveness of NGS-guided management strategies in OME.

Such work is essential to determine which probiotic strains, dosages and delivery routes yield meaningful clinical benefits in the management of OME [57].

## 6. Limitations

In this review, keywords were used in order to find relevant literature. The search was not based on a specific protocol and thus may have resulted in an omission of relevant studies. There is a lack of a healthy control group in certain articles. This limits the interpretation of whether the reported microbial profiles are specific to OME or reflect background microbiota, transient colonization, or methodological contamination.

Furthermore, DNA sequencing can identify the presence of the bacteria yet cannot reveal its activity or viability [25,48]. 16S rRNA sequencing may not detect bacteria below the genus level encompassing respiratory pathogens and commensals [25].

Another important limitation relates to the heterogeneity of research findings regarding the middle ear microbiome in children with OME. This variability may stem from differences in age, comorbidities, environmental factors, and prior antibiotic treatment. A major limitation is the general lack of key clinical parameters such as age, antibiotic history, and effusion duration, which prevents a clear correlation between the microbiome and the patient’s clinical history across studies. Another variable is the differences in sample types and collection sites. Methodological differences in next-generation sequencing techniques may further contribute to the inconsistent findings. When interpreting microbiome data, the possibility of contamination in samples with low biomass must be taken into account. In this context, strict aseptic sampling is particularly important, especially to minimize potential contamination from the external auditory canal during middle ear fluid collection [22,24]. Differences in DNA extraction protocols, primer selection, targeted 16S rRNA hypervariable regions, and sequencing platforms may also influence the detected microbial profile and taxonomic resolution [58]. Moreover, variation in bioinformatics pipelines, reference databases, and statistical approaches used for alpha- and beta-diversity analyses, including whether false discovery rate correction was applied for multiple comparisons, may limit direct comparability between studies [59].

As this work is a narrative review, the presented synthesis should be interpreted as descriptive rather than quantitative. The simplified tables were used to improve clarity and avoid inappropriate comparison of heterogeneous microbiome metrics, including relative abundance, detection frequency, PCR positivity, culture positivity, and prevalence. Consequently, the tables and heatmap identify recurrent patterns across studies but do not provide pooled estimates or meta-analytic conclusions.

## 7. Conclusions

The reviewed studies lead to the conclusion that otitis media with effusion (OME) is closely associated with microbiota dysbiosis and bacterial biofilm formation. Taken together, current evidence supports a shift from viewing OME as a purely sterile inflammatory process toward recognizing it as a condition potentially influenced by microbial dysbiosis, biofilm formation, and host–microbiome interactions. However, due to the low-biomass nature of the middle ear and methodological heterogeneity across studies, further research is necessary to confirm these findings and definitively rule out contamination biases. Current evidence suggests that the adenoids and nasopharynx may act as potential microbial reservoirs, although several studies also indicate substantial niche-specific differences between these sites and the middle ear. Therefore, given the inefficacy of antibiotic therapy, microbiome modulation via targeted probiotics represents a promising area for future research, though current evidence is insufficient to recommend it as a replacement for established surgical treatments.

Furthermore, attention must be drawn to molecular diagnostic methods. NGS may complement conventional culture by enabling the detection of fastidious or previously unrecognized bacteria, although its clinical interpretation requires caution. In contrast, traditional culture-based techniques frequently give false-negative results or fail to detect dominant pathogens.

## Figures and Tables

**Table 1:** Characteristics and classification of microbiome studies included in the qualitative synthesis.

Study	Country	Population/Design	Sample Type	Sequencing/Molecular Method	16S Region	Bioinformatics/Analysis
Jervis-Bardy et al. 2015 [37]	Australia	Indigenous children with OME	MEF, nasopharynx, adenoid	16S rRNA amplicon sequencing	V3–V4	QIIME; OTU-based analysis; diversity and beta-diversity analyses
Chan et al. 2016 [29]	Australia	Children with chronic OME	MEF, external auditory canal, adenoid	16S rRNA amplicon sequencing	V3–V4	QIIME; Greengenes; relative abundance and diversity analyses
Krueger et al. 2017 [55]	USA	Children with chronic otitis media/OME	MEF	16S rRNA amplicon sequencing	V4	mothur/QIIME; SILVA; diversity and differential abundance analyses
Boers et al. 2018 [52]	Netherlands	Children with OM with/without GER tendency	MEF, nasopharynx	16S rRNA gene sequencing	V5–V6	mothur; low-biomass filtering; paired MEF-NP analysis
Lappan et al. 2018 [23]	Australia	Case–control study of recurrent AOM	MEF, middle ear rinse, nasopharynx, ear canal	16S rRNA amplicon sequencing	V3–V4	UPARSE/QIIME; SILVA; differential abundance and niche analyses
Walker et al. 2019 [24]	New Zealand	Case–control study of chronic OME vs. controls	Anterior nasal swabs	16S rRNA amplicon sequencing	V1–V3	USEARCH/UPARSE; Bray–Curtis; DESeq2; clustering
Kolbe et al. 2019 [54]	USA	Re-analysis of MEF samples from children with COME	MEF	16S rRNA ASV re-analysis	V4	DADA2; SILVA; PICRUSt2; DESeq2; UniFrac
Ari et al. 2019 [38]	Turkey	Children with OME undergoing VT and/or adenoidectomy	MEE, adenoid tissue	Ion Torrent 16S metagenomics	V2, V3, V4, V6–V7, V8, V9	Ion Reporter; Greengenes/MicroSEQ; QIIME diversity metrics
Kim et al. 2019 [36]	Republic of Korea	Children with OME vs. controls	Adenoid samples	16S rRNA sequencing	V3–V4	QIIME; UniFrac; OTU/alpha diversity analyses
Xu et al. 2020 [56]	China	Children with OME and adenoid hypertrophy plus controls	MEE, adenoid swabs	16S rRNA sequencing	V4	OTU-based diversity and beta-diversity analyses
Jörissen et al. 2021 [57]	Belgium	Case–control microbiome study of chronic OME	MEF, nasopharynx, ear canal, adenoid	16S rRNA amplicon sequencing	V4	ASV-based workflow; contaminant filtering; differential abundance; culture follow-up
Huang et al. 2021 [58]	Taiwan	Children with OME or SDB undergoing adenoidectomy	Adenoid tissue	16S rRNA sequencing	V3–V4	QIIME2; Bray–Curtis; DESeq2; pneumococcal carriage analysis
Sokolovs-Karijs et al. 2024 [59]	Latvia	Children with OME vs. children with healthy middle ears	Adenoid surface swabs	16S rRNA sequencing	V3–V4	QIIME2/DADA2; SILVA; alpha/beta diversity; contaminant-aware workflow
Fekete et al. 2024 [7]	Hungary	Children with OME	MEF	Culture plus 16S rRNA sequencing	V3–V4	CosmosID; Shannon/Chao; Bray–Curtis; PERMANOVA; LEfSE
Göçer et al. 2025 [60]	Turkey	Children with OME and controls without OM	MEC, nasopharynx	16S rRNA sequencing	V3–V4	QIIME2/DADA2; SILVA 138; ASV-based workflow

AOM—acute otitis media; ASV—amplicon sequence variant; COME—chronic otitis media with effusion; DADA2—Divisive Amplicon Denoising Algorithm 2; GER—gastroesophageal reflux; LEfSE—linear discriminant analysis effect size; MEC—middle ear cavity; MEE—middle ear effusion; MEF—middle ear fluid; NP—nasopharynx; OM—otitis media; OME—otitis media with effusion; OTU—operational taxonomic unit; PERMANOVA—permutational multivariate analysis of variance; PICRUSt2—Phylogenetic Investigation of Communities by Reconstruction of Unobserved States 2; QIIME—Quantitative Insights Into Microbial Ecology; SDB—sleep-disordered breathing; SILVA—ribosomal RNA gene database; UPARSE—USEARCH-based sequence analysis pipeline; USA—United States of America; VT—ventilation tube; 16S rRNA—16S ribosomal ribonucleic acid; DESeq2—Differential Expression Analysis for Sequence Count Data 2; QIIME2—Quantitative Insights Into Microbial Ecology 2; USEARCH—unique sequence analysis software; UniFrac—unique fraction metric.

**Table 2:** Frequently reported bacterial genera in pediatric OME microbiome studies.

Genus/Taxon	Main Sample Source(s)	Consistency Across Studies	Interpretation in OME
*Alloiococcus*	MEF/MEE/MEC; ear canal	Very high in MEF-focused studies	Recurrent pathobiont candidate; may dominate low-diversity MEF communities; possible ear canal contribution in some studies
*Haemophilus*	MEF, nasopharynx, adenoid	Very high	Classical otopathogen; associated with AOM/OME continuum and mucin/inflammatory responses
*Moraxella*	Nasopharynx, adenoid, MEF	Moderate to high	Classical upper airway otopathogen; more prominent in NP/adenoid than some MEF datasets
*Streptococcus*	Nasopharynx, adenoid, MEF	High	Includes classical pathogens and commensal/protective species; genus-level interpretation is limited
*Staphylococcus*	MEF, ear canal, adenoid	Moderate	Possible pathobiont, contaminant, or ear canal-associated signal depending on sampling context
*Turicella*/*Corynebacterium*	MEF, ear canal	Moderate	Ear canal-associated taxa; possible pathobiont role remains debated
*Pseudomonas*	MEF, ear canal	Low to moderate	Opportunistic genus; inconsistently reported; may reflect ecology, contamination, or regional effects
*Prevotella*/*anaerobes*	Adenoid, nasopharynx, oral-associated niches	Moderate in adenoid studies	Supports polymicrobial/anaerobic and oral-adenoid ecological contribution
*Fusobacterium*/*Peptostreptococcus*	Adenoid, oral-associated niches	Moderate in recent adenoid studies	May reflect anaerobic adenoid ecology and possible oral-nasopharyngeal contribution
*Sphingobium*	MEF in recent studies	Emerging/recent	Detected in recent MEF sequencing studies; interpretation uncertain
*Dolosigranulum*/*Lactobacillus*/*Propionibacterium*	Nasal/nasopharyngeal or control-associated microbiota	Supportive protective signal	Potential protective or health-associated taxa in upper airway studies

AOM—acute otitis media; MEC—middle ear cavity; MEE—middle ear effusion; MEF—middle ear fluid; NP—nasopharynx; OME—otitis media with effusion.

**Table 3:** Qualitative overview of middle ear effusion/middle ear cavity microbiome findings.

Study	Dominant/Recurrent MEF Taxa	Additional Reported Taxa	Diversity/Ecology Findings
Jervis-Bardy et al. 2015 [37]	*Alloiococcus*, *Haemophilus*	*Streptococcus*, *Moraxella*, *Corynebacterium*/*Turicella*	MEF showed low diversity and single-OTU dominance in many samples; MEF differed from NP/adenoid microbiota
Chan et al. 2016 [29]	*Alloiococcus*, *Haemophilus*	*Moraxella*, *Staphylococcus*, *Streptococcus*, *Corynebacterium*	MEF microbiota partly overlapped with EAC and adenoid niches; high inter-patient variability
Krueger et al. 2017 [55]	*Haemophilus*	*Moraxella*, *Turicella*, *Pseudomonas*, *Alloiococcus*	Clinical variables including age, hearing loss, and mucin profile were associated with microbiome differences
Boers et al. 2018 [52]	*Alloiococcus*, *Turicella*	*Haemophilus*, *Streptococcus*, *Staphylococcus*	MEF and nasopharyngeal microbiota were site-specific; low-DNA samples were filtered
Lappan et al. 2018 [23]	*Alloiococcus*, *Haemophilus*	*Staphylococcus*, *Turicella*, *Pseudomonas*, *Streptococcus*	MEF, middle ear rinse, NP, and ear canal showed compartment-specific communities
Kolbe et al. 2019 [54]	*Haemophilus*, *Moraxella*, *Alloiococcus*	*Staphylococcus*, *Turicella*	Lower-airway disease status associated with altered MEF diversity and differential abundance
Ari et al. 2019 [38]	*Alloiococcus*	*Turicella*, *Staphylococcus*	MEE bacteriome differed from adenoid bacteriome; OME interpreted as polymicrobial
Xu et al. 2020 [56]	*Haemophilus*, *Staphylococcus*	*Halomonas*, *Streptococcus*, *Moraxella*	MEF microbiome structure was dissimilar to adenoid microbiome by beta-diversity analyses
Jörissen et al. 2021 [57]	*Haemophilus*, *Alloiococcus*	*Moraxella*, *Streptococcus*, *Turicella/Corynebacterium*, *Staphylococcus*	Many effusions were dominated by one ASV; *Alloiococcus*/*Turicella*/*Staphylococcus* signals often resembled ear canal profiles
Fekete et al. 2024 [7]	*Alloiococcus*	*Haemophilus*, *Streptococcus*, *Sphingobium*, *Moraxella*, *Corynebacterium*	High-*Alloiococcus* samples showed significantly lower alpha and beta diversity; culture and sequencing were discordant
Göçer et al. 2025 [60]	*Alloiococcus*	*Haemophilus*, *Streptococcus*, *Corynebacterium*, *Moraxella*, *Staphylococcus*, *Sphingobium*	OME MEC samples showed enrichment of *Alloiococcus* and reduction in potentially protective genera; controls included healthy middle ear cavity samples

ASV—amplicon sequence variant; EAC—external auditory canal; MEC—middle ear cavity; MEE—middle ear effusion; MEF—middle ear fluid; NP—nasopharynx; OME—otitis media with effusion; OTU—operational taxonomic unit; DNA—deoxyribonucleic acid.

**Table 4:** Qualitative overview of adenoid microbiome findings relevant to pediatric OME.

Study	Sample Type	Main Taxa/Patterns	Diversity Findings
Jervis-Bardy et al. 2015 [37]	Adenoid swabs	*Haemophilus*, *Streptococcus*, *Moraxella*, *Prevotella*/anaerobic taxa	Adenoid communities were richer and more diverse than MEF
Ari et al. 2019 [38]	Adenoid tissue	*Rothia*, *Veillonella*, *Granulicatella*, *Prevotella*, *Staphylococcus*	Adenoid bacteriome differed from MEE despite paired sampling
Kim et al. 2019 [36]	Adenoid samples	*Haemophilus*, *Streptococcus*, *Prevotella*, *Corynebacterium*	OME group showed reduced diversity compared with controls
Xu et al. 2020 [56]	Adenoid swabs	*Haemophilus*, *Streptococcus*, *Moraxella*, *Neisseria*	Adenoid microbiota in OME and controls were similar; MEF differed from adenoids
Huang et al. 2021 [58]	Adenoid tissue	*Alloprevotella*, *Staphylococcus*, *Moraxella*, *Neisseriaceae in pneumococcal* carriage-positive samples	Pneumococcal carriage associated with lower diversity
Sokolovs-Karijs et al. 2024 [59]	Adenoid surface swabs	*Haemophilus*, *Fusobacterium*, *Streptococcus*, *Moraxella*, *Peptostreptococcus*; OME enriched in *Fusobacterium*/*Peptostreptococcus* and anaerobic genera	Healthy-ear group had greater evenness; OME group showed greater beta-diversity variability

MEF—middle ear fluid; MEE—middle ear effusion; OME—otitis media with effusion.

**Table 5:** Nasal and nasopharyngeal microbiome evidence relevant to pediatric OME.

Study	Sample Type	Main Findings	Health-Associated/Protective Signal
Walker et al. 2019 [24]	Anterior nasal swabs	Children with chronic OME had lower nasal diversity and higher abundance of otopathogen-associated profiles	Mixed nasal profile, alpha-hemolytic streptococci, Lactococcus, Propionibacterium
Lappan et al. 2018 [23]	Nasopharyngeal swabs	rAOM cases and controls had distinct NP microbiomes; cases had more diverse and pathobiont-associated profiles	*Corynebacterium* and *Dolosigranulum* were enriched in controls
Boers et al. 2018 [52]	Nasopharyngeal swabs	*Haemophilus* and *Streptococcus* in MEF were detected when the same genera were present in NP in paired samples	No clear GER-related NP/MEF microbial effect
Jörissen et al. 2021 [57]	Nasopharyngeal swabs	OME and control NP microbiomes differed; no single OME-specific NP pathogen dominated	*Streptococcus salivarius* group and *Acinetobacter lwoffii* were health-associated
Göçer et al. 2025 [60]	Nasopharyngeal samples from OME group	Compared NP and MEC microbiota in OME context	Potentially protective genera reduced in OME-associated middle ear samples

MEC—middle ear cavity; MEF—middle ear fluid; NP—nasopharynx; OME—otitis media with effusion; rAOM—recurrent acute otitis media; GER—gastroesophageal reflux.

**Table 6:** Biofilm, targeted molecular, and non-core mechanistic studies.

Study	Methodology	Main Finding
Hall-Stoodley et al. 2006 [61]	Middle-ear mucosa biopsy; CLSM, FISH, immunostaining, PCR/culture of effusions	Biofilms were directly visualized on middle-ear mucosa in chronic/recurrent OM
Topcuoglu et al. 2012 [41]	Targeted molecular/clonality analysis	*Fusobacterium nucleatum* and *Treponema denticola* findings support oral-nasopharyngeal-middle-ear translocation hypothesis
Khoramrooz et al. 2012 [62]	Targeted PCR/PFGE-type pathogen study	Species-specific detection/typing rather than microbiome ecology
Emami et al. 2019 [63]	Culture plus targeted PCR panel	Reported regional *Pseudomonas aeruginosa* detection in MEF

CLSM—confocal laser scanning microscopy; FISH—fluorescence in situ hybridization; MEF—middle ear fluid; PCR—polymerase chain reaction; PFGE—pulsed-field gel electrophoresis; OM—otitis media.

**Table 7:** Methodological heterogeneity across included microbiome studies.

Source of Heterogeneity	Examples Across Included Studies	Potential Impact
Different 16S rRNA hypervariable regions	V1–V3, V3–V4, V4, V5–V6, and multi-region Ion Torrent panels	Taxonomic bias; inconsistent detection of genera/species
Different sequencing platforms	Illumina MiSeq, Ion Torrent, re-analysis datasets	Platform-specific read length and error profiles
Different DNA extraction methods	Commercial kits, CTAB, bead beating, soil kits for swabs	Differential lysis of Gram-positive organisms and low-biomass signals
Different bioinformatic pipelines	QIIME, mothur, UPARSE, DADA2, CosmosID, Ion Reporter	OTU/ASV resolution and taxonomic assignments differ
Different reference databases	SILVA, Greengenes, MicroSEQ, NCBI-based assignment	Misclassification risk, especially for closely related taxa
Low biomass of MEF	MEF samples often have low bacterial load and variable sequencing depth	Increased risk of reagent/environmental contamination and stochastic dominance
Sample-type differences	MEF/MEE/MEC vs. adenoid tissue/swabs vs. NP/nasal vs. ear canal	Anatomical niches are not interchangeable
Different reporting metrics	Relative abundance, detection frequency, prevalence, culture positivity	Values may appear contradictory or sum above 100% if mixed
Clinical heterogeneity	OME, COME, rAOM; variable prior surgery, GER, respiratory disease, age, antibiotics	Population-specific microbiome differences
Culture vs. sequencing discordance	Fekete 2024 [7] and other studies show non-concordance	Culture may miss fastidious taxa; sequencing may detect nonviable DNA

ASV—amplicon sequence variant; COME—chronic otitis media with effusion; CTAB—cetyltrimethylammonium bromide; DADA2—Divisive Amplicon Denoising Algorithm 2; DNA—deoxyribonucleic acid; GER—gastroesophageal reflux; MEC—middle ear cavity; MEE—middle ear effusion; MEF—middle ear fluid; NCBI—National Center for Biotechnology Information; NP—nasopharynx; OME—otitis media with effusion; OTU—operational taxonomic unit; QIIME—Quantitative Insights Into Microbial Ecology; rAOM—recurrent acute otitis media; SILVA—ribosomal RNA gene database; UPARSE—USEARCH-based sequence analysis pipeline; 16S rRNA—16S ribosomal ribonucleic acid.

## Data Availability

No new data were created or analyzed in this study.

## Abbreviations

The following abbreviations are used in this manuscript:

16S rRNA	16S ribosomal ribonucleic acid
AOM	acute otitis media
ASV	amplicon sequence variant
CFU	colony-forming units
CLSM	confocal laser scanning microscopy
COME	chronic otitis media with effusion
CTAB	cetyltrimethylammonium bromide
DADA2	Divisive Amplicon Denoising Algorithm 2
DESeq2	Differential Expression Analysis for Sequence Count Data 2
DNA	deoxyribonucleic acid
EAC	external auditory canal
FISH	fluorescence in situ hybridization
GER	gastroesophageal reflux
GERD	gastroesophageal reflux disease
LEfSE	linear discriminant analysis effect size
LGG	*Lactobacillus rhamnosus* GG
*M. catarrhalis*	*Moraxella catarrhalis*
MEC	middle ear cavity
MEE	middle ear effusion
MEF	middle ear fluid
NCBI	National Center for Biotechnology Information
NGS	next-generation sequencing
NICE	National Institute for Health and Care Excellence
NP	nasopharynx
OM	otitis media
OME	otitis media with effusion
OTU	operational taxonomic unit
PCV	pneumococcal conjugate vaccine
PCV10	10-valent pneumococcal conjugate vaccine
PCR	polymerase chain reaction
PERMANOVA	permutational multivariate analysis of variance
PFGE	pulsed-field gel electrophoresis
PICRUSt2	Phylogenetic Investigation of Communities by Reconstruction of Unobserved States 2
PRISMA	Preferred Reporting Items for Systematic Reviews and Meta-Analyses
PRISMA-ScR	Preferred Reporting Items for Systematic Reviews and Meta-Analyses extension for Scoping Reviews
qPCR	quantitative polymerase chain reaction
QIIME	Quantitative Insights Into Microbial Ecology
rAOM	recurrent acute otitis media
ROM	recurrent otitis media
SDB	sleep-disordered breathing
SILVA	ribosomal RNA gene database
UPARSE	USEARCH-based sequence analysis pipeline
USA	United States of America
UniFrac	unique fraction metric
VT	ventilation tube
